# In vitro-antibacterial properties of ten medicinal plants against common uropathogenic organisms and toxicity determination using brine shrimp lethality assay

**DOI:** 10.1186/s12906-024-04595-y

**Published:** 2024-08-14

**Authors:** Jayananda Ilangage Maheshi Kavindya Ilangage, Ilangakoon Arachchige Chethana Sewwandi Ilangakoon, Kariyakarawanage Minoshi Kaushika Fernando, Dissanayaka Mudiyanselage Manisha Kavindu Dissanayake, Nimna Pinimali Deeyagaha Rajapaksha, Lakmini Hasanthika Walpola, Dineshani Hettiarachchi

**Affiliations:** 1 Department of Biomedical Science, Faculty of Health Science, Kaatsu International University (KIU), Colombo, Sri Lanka; 2 Department of Anatomy, Genetics and Biomedical Informatics, Faculty of Medicine, University of Colombo, Colombo, Sri Lanka

**Keywords:** Antimicrobial, BSLA, Medicinal plant, Uropathogenic, UTIs

## Abstract

**Background:**

In recent years, antibiotic resistance has emerged as a global health concern in bacterial infections such as urinary tract infections (UTIs). Uropathogenic *Escherichia coli* is the most frequent organism responsible for both simple and complex UTIs. *Staphylococcus aureus* and *Pseudomonas aeruginosa* are frequently associated with complicated UTIs. Sri Lanka has significant resources of medicinal plants used to cure UTIs in Ayurvedic and traditional medicine.

**Methods:**

Agar well diffusion and broth microdilution methods were used to determine the antibacterial activity of the methanolic extract of ten medicinal plants against *P. aeruginosa* ATCC27853, *S.aureus* ATCC25923, *E.coli* ATCC25922 and their UTI positive strains extracted from positive culture plates. As a preliminary toxicity assay, the Brine Shrimp Lethality Assay (BSLA) was used to determine its cytotoxicity.

**Results:**

The methanolic fruits extract of *P. emblica* demonstrated the highest antibacterial activity against both *E. coli* ATCC25922 and *E. coli* UTI-positive strains. *B. diffusa* roots extract exhibited the highest activity against *S. aureus* ATCC25923, while *T. chebula* fruits extract showed the highest activity against the *S. aureus* UTI-positive strain. *T. involucrata* roots extract displayed the highest activity against *P. aeruginosa* ATCC27853, and *Z. officinale* rhizomes extract showed the highest activity against the *P. aeruginosa* UTI-positive strain. Moreover, the plant mixture showed the most substantial antibacterial effect against *P. aeruginosa* ATCC27853. However, the methanolic seed extract of *C. melo* did not exhibit any antimicrobial effects against the selected organisms. All plant material, including the plant mixture, showed cytotoxicity according to the BSLA.

**Conclusion:**

All the methanolic extracts including *P. emblica* fruits, *O. tenuiflorum* whole plant, *T. chebula fruits*,* Z. officinale rhizome*,* T. terrestris* roots, *T. involucrata* roots, *A. lanata* whole plant. *B. diffusa* roots and *A. falcatus* roots showed antimicrobial effects against selected strains except *C. melo* seed extract. The results of the present study evidently supports the traditional and ayurvedic use of these plants for the treatment of UTIs. This paves the way for another praise for new plant-based therapeutic product development for the treatment of UTIs. However, further toxicity studies are needed for medicinal dose determination.

## Background

Urinary tract infections (UTIs) are the commonest form of bacterial infections, affecting people throughout their live cycle. They can affect up to 150 million individuals worldwide annually [1]. Both complicated and simple UTIs have a complex pathogenesis influenced by a variety of biological, behavioral, and specific uropathogenic host variables. Uropathogenic *Escherichia coli* is the most frequent organism underpinning both simple and complex UTIs (UPEC). *Staphylococcus aureus* and *Pseudomonas aeruginosa* are mainly associated with complicated UTIs [2].

Antimicrobial drugs are widely misused, which has caused bacterial infections to evolve from being easily curable to that more resistant. Despite the availability of powerful antibiotics, resistant or multidrug-resistant bacteria continue to emerge, necessitating the search for and creation of novel treatment modalities. Plants have been utilized worldwide for centuries to treat infections. These herbal compounds can act as prototypes for drug development while being efficient and less harmful to the host [3]. There is a large range of secondary metabolites that medicinal plants produce, many of which have been found to have therapeutic potential and are a promising source to help combat drug resistance. Multiple mechanisms are thought to be involved in antibacterial activity of some plants owing to their various functional groups and additional antioxidant activity [4]. For example, *Ocimum tenuiflorum* leaves are rich in volatile oil phenolics, flavonoids, neolignans, terpenoids and fatty acid derivatives and their seeds contain fixed oil mucilage, polysaccharides and β-sitosterol in the unsaponifiable matter. Seed oil, which is rich in triglycerides in linolenic acid, is the main content. Flavonoids including orientin and vicenin were screened against bacterial strains causing UTIs in humans such as *Staphylococcus aureus*,* Staphylococcus cohni* (gram-positive), *Escherichia coli*, *Proteus*, *Klebsiella pneumonia* (gram-negative). The synergistic effect of orientin and vicenin on antibacterial activity showed better results in all the strains than individual flavonoids with maximum concentrations against *E. coli*,* Proteus*,* S. aureus*,* S. cohni* and *K. pneumonia*, respectively [5]. Few reports declare that the methanol extract of *A. lanata* gives rise to a significant number of medicinally beneficial phytochemicals including kaempferol, isorhamnetin, quercetin, and flavanone are dominant phytochemicals that belong to flavonoid and contain trace of apigenin, ferulic acid, syringic acid, narcissin, and vanillic acid. Due to the presence of abundant phytochemical compounds, *A. lanata* plant has been used in indigenous medicine for several decades. Plants that grow in an evergreen region with minerals-abundant soil may have a higher concentration of medicinally valuable phytochemical constituents. Their effectiveness against opportunistic pathogens are yet to be demonstrated [6].

According to the World Health Organization (WHO), over 80% of the world’s population relies on traditional medicine for their main healthcare requirements [7, 8]. Additionally, more than 50% of all clinically used medicine today have originated from natural products [9]. In more than 80% of developed countries, plants have been used as traditional medicine as they are a good source of compound derivation. Many plants have been used for their antimicrobial traits, which are chiefly due to the synthesis of secondary metabolites and their inhibitory effect against the growth of human pathogens [10]. Sri Lanka has a rich source of medicinal plants as it harbors over 3000 plant species [11]. Therefore, this study aims to develop a plant-based treatment for uropathogenic bacterial infections (*Escherichia coli*,* Staphylococcus aureus and Pseudomonas aeruginosa)* and to determine its synergetic effects against the selected organisms including their cytotoxicity, using the Brine Shrimp Lethality Assay (BSLA). We selected ten herbal medicinal plants *(Phyllanthus emblica*,* Ocimum tenuiflorum*,* Terminalia chebula*,* Zingiber officinale*,* Tribulus terrestris*,* Cucumis melo*,* Tragia involucrata*,* Aerva lanata. Boerhavia diffusa* and *Asparagus falcatus****)*** in consultation with the opinion of the Ayurvedic medical practitioners and following a thorough literature review. The selected plants were known to possess antibacterial properties and many Ayurvedic practitioners use these plants to treat UTIs and urinary tract-related diseases. However, the antimicrobial properties of these plants and most of the extractions were based on aqueous and alcoholic extraction methods. Hence, we selected the methanolic extraction method to enhance the solubility of the plant compounds. Methanol has been found to be more efficient in extracting most of the phytochemicals and low molecular weight polyphenols. Preparation of the plant extract will open new avenues for extraction and can aid in the development of new formulations according to their dose and toxicity [12].

## Methods

### Plant material collection

**Fig. 1:**
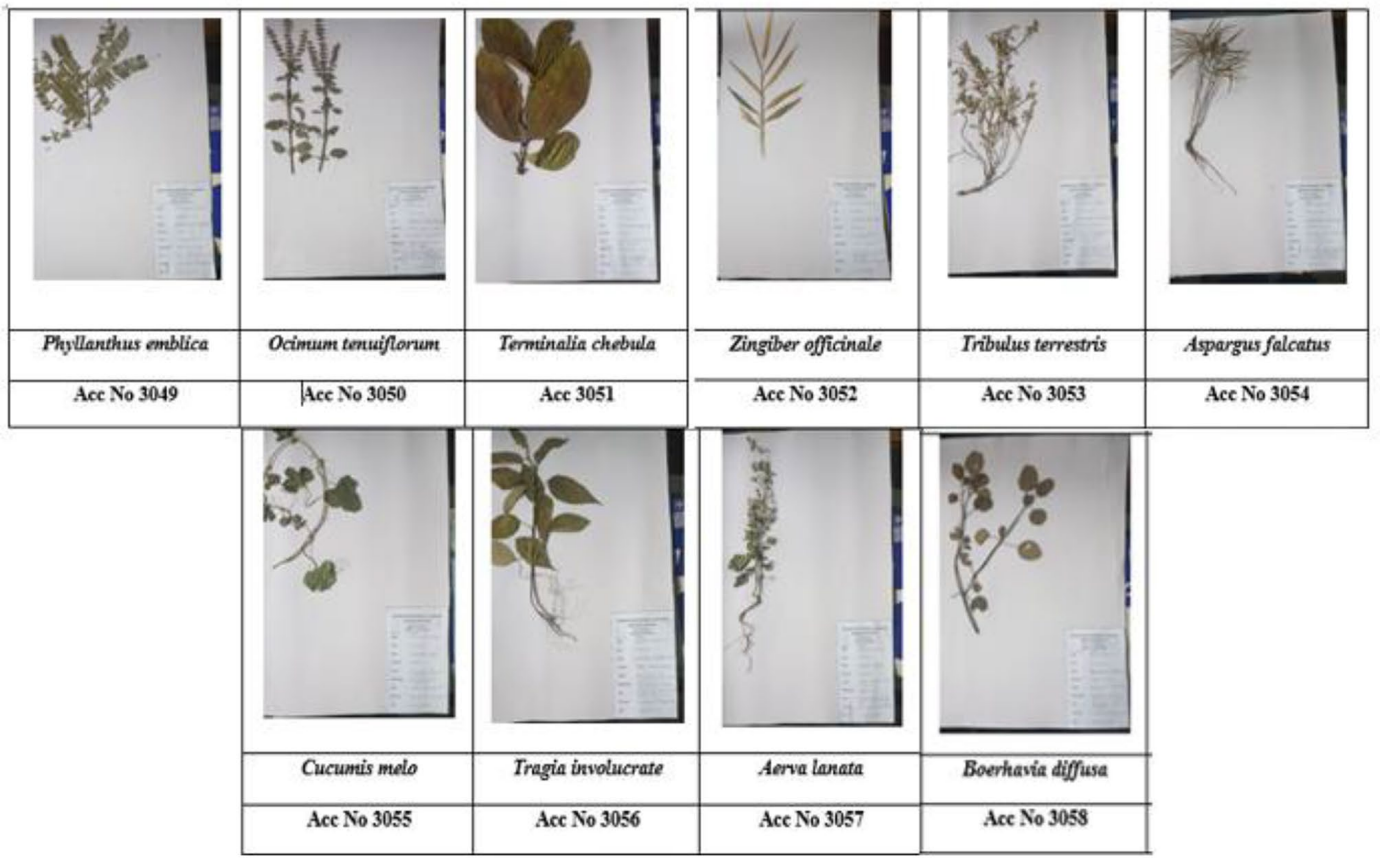
Plants’ voucher specimens with their reference numbers

Medicinal plants were identified based on the literature review and expert opinion of Ayurvedic and traditional medicinal practitioners on treatment of UTIs. Table 1 includes the ten selected medicinal plants with their selected parts chosen for the present study and the plant authenticated reference numbers. Except *T. terrestris*, all other plants were harvested. The *T. terrestris* plants were collected from the coastal areas of Sri Lanka. Plant authentication for all ten plants were obtained from the Botany Division, Bandaranayake Memorial Ayurvedic Research Institute, Nawinna, Maharagama, Sri Lanka by Ms. Pushpa Jeewandara (Scientific officer / Pharmacognocy). Voucher specimen of the plant material has been deposited in the Botany Division, Bandaranayake Memorial Ayurvedic Research Institute, Nawinna, Maharagama, Sri Lanka. Pictures of the voucher specimens and the reference numbers are included in the Fig. 1. All the plant material were freshly collected in bulk and dried at room temperature (20-30^o^C) and the dried plant material were sealable bags and the plant materials were transferred to the Biomedical Science laboratory, KIU, Sri Lanka.

**Table 1:** Profile of authenticated plants and their selected parts used in the present study

Acc. No/ Reference No	Local name in English	Local name in Sinhala	Scientific name	Family	Extraction part
3049	Amla	Beheth nelli/ nelli	*Phyllanthus emblica*	EUPHORBIACEAE	Fruit
3050	Holy basil/ Sacred basil/ Tulsi	Heen maduruthala	*Ocimum tenuiflorum*	LAMIACEAE	Whole plant
3051	Chebulic Myrobalan/ Gall nut	Aralu	*Terminalia chebula*	COMBRETACEAE	Fruit
3052	Ginger	Inguru	*Zingiber officinale*	ZINGIBERACEAE	Rhizome
3053	Land Calthrop	Heen nerenchi	*Tribulus terrestris*	ZYGOPHYLLACEAE	Root
3054	Satawari	Hathawariya	*Asparagus falcatus*	ASPARAGACEAE	Root
3055	Muskmelon	Kakiri	*Cucumis melo*	CUCURBITACEAE	Seeds
3056	Climbing Nettle	Wel kahabiliya	*Tragia involucrata*	EUPHORBIACEAE	Root
3057	Mountain Knotgrass	Polpala	*Aerva lanata*	AMARANTHACEAE	Whole plant
3058	Hog weed	Sarana/ pita sudu sarana/ beth sarana	*Boerhavia diffusa*	NYCTAGINACEAE	Root

Medicinal plants were selected according to the advice of Ayurvedic and traditional medicinal practitioners and according to the literature review

### Preparation of plant material extract using methanol

The process followed the method described by Ahmed et al. [13]. The fresh weight of the plant material was measured, and they were dried in the shade or oven-dried. It was ground to obtain a fine powder by using a blender [14]. Ten grams of the prepared sample was dissolved in 100 mL of 99% methanol by vigorous shaking for 10 min. It was rested for 48 h at room temperature. Following which the crude extract was filtered, the solvents evaporated (45–50 °C) and extracts were kept for further study [13].

### Collection of bacterial samples

The bacterial strains used were isolated from UTI-positive culture plates and standard reference bacteria were used for the following three bacterial strains; *Escherichia coli* ATCC25922, *Staphylococcus aureus* ATCC25923 and *Pseudomonas aeruginosa* ATCC27853 according to the CLSI guidelines [15].

### Antibacterial activity assays

Antibacterial activities of the methanol extracts of the ten plants were tested against microorganisms isolated from positive UTI culture plates and standard ATCCs. In vitro antibacterial activity was tested in the presence or absence of a zone of inhibition, the minimum inhibitory concentration (MIC) and minimum bactericidal concentration (MBC) was determined using Gentamycin as the reference. The diameter of the ZOI was measured to the nearest mm and the mean Zone of Inhibition (ZOI) was calculated. Relative percentage inhibition and the standard deviation for each plant extract were calculated using Microsoft Excel software and the formula stated below. Relative percentage inhibition was calculated with respect to the positive control, Gentamycin (10$\:\mu\:$g/ml).

$$\>{\rm{Mean}}\>{\rm{ZOI}} = {{{\rm{ZO}}{{\rm{I}}_1} + {\rm{ZO}}{{\rm{I}}_2} + {\rm{ZO}}{{\rm{I}}_3}} \over {\rm{N}}}$$

ZOI_1_ = Zone of inhibition measured on culture plate 1 for a given concentration.

ZOI_2_ = Zone of inhibition measured on culture plate 2 for a given concentration.

ZOI_3_ = Zone of inhibition measured on culture plate 3 for a given concentration.

n = Number of tests performed (The test was performed in triplicates. Therefore, *n* = 3).

$${\rm{Relative}}\,{\rm{percentage}}\,{\rm{inhibition}}\>\left( {{\rm{RPI}}} \right)\% {\rm{ = }}\>{{({\rm{a - c}})\> \times \>{\rm{100}}} \over {(b{\rm{ - c}})}}$$

a = mean ZOI of the tested plant extract in mm.

b = mean ZOI of the positive control in mm.

c = mean ZOI of the negative control in mm [6].

#### Agar well diffusion assay

The Kirby-Bauer technique was used to determine the antibacterial activity of the plant extracts. Mueller Hinton agar (pH 7.2- & 4-mm depth) plates were inoculated with test organisms by streaking the loop in a back-and-forth motion to ensure an even distribution of inoculum according to the 0.5 McFarland turbidity [16]. Five wells of 6 mm diameter were cut in the medium with the help of a sterile 100 µl pipette tip. 50 mg/ml working solution for each plant material was prepared using methanolic extract and 50% Dimethyl sulfoxide (DMSO), from this mixture 100 µl were added to each well [17]. The same volume was used for the control. In addition, the reference antibiotic was added into a well on each plate. Plates were left for some time till the extract diffused into the medium and incubated at 37 °C for 24 h. After incubation, plates were observed for the zone of inhibition. Sterility was maintained throughout the procedure [18].

#### Broth microdilution assay

The minimum inhibitory concentration (MIC) was tested using the broth dilution method. Ten tubes were prepared each containing 2 ml of Muller Hinton agar broth (MHB) medium and numbered from 1 to 20, 2 ml of plant extract stock solution was transferred and placed in tube 1 and shaken well to get a concentrate. Then 2 ml was transferred from this tube to the second tube and repeated for all tubes, 0.1mL of pre-prepared bacterial suspension was transferred to each tube and shaken for homogenization of the bacteria. The tubes were incubated at 37C^o^ for 24 h [17, 19].

#### Minimum bacterial concentration (MBC)

The last tubes with no growth in MIC assay were sub-cultured on nutrient agar (NA) plates and incubated at room temperature for 24 h. The lowest concentration that killed 100% of the inoculum bacteria (no growth on the plate) was recorded as the Minimum Bactericidal Concentration [20].

### Brine shrimp lethality assay (BSLA)

Brine shrimp (*Artemia salina*) lethality assay is an important tool for the preliminary cytotoxicity assay of plant extract and others based on the ability to kill a cultured larva (nauplii). Larvae were exposed to different concentrations of plant extract for 24 h. Then the number of motile larvae (alive larvae) were calculated for the effectiveness of the extract and the LD50 value using Graph Pad Prism 9 software [21]. This is an inexpensive screening technique mainly performed to assess the toxic nature of plant extracts and their derivative compounds. Filtered, seawater (pH range; 7.85 ± 0.5) was added in a small hatching chamber with a partition for dark (covered) and light supply. Brine Shrimp eggs (0.5 g) were added into the dark side of the chamber. The eggs were hatched for 24–36 h at room temperature [22]. 1 mg/ ml stock solution was prepared (the stock solution prepared by dissolving 10 mg of plant extract in 10 ml of 1% DMSO). Concentrations of 1 mg/ ml, 100 µg/ ml, 10 µg/ ml, and 1 µg/ mL were prepared by serial dilution from the stock solution. Five test tubes each containing 1 mL of solution,10 live nauplii of *A. salina* and 1 ml of seawater, were labeled 1 to 5 and 1% DMSO without extract was used as a negative control. The number of dead and live nauplii were counted after incubating for 24 h at 25 ^o^C [21]. The results were recorded as the percentage of mortality after 24 h and 50% Lethal Concentration (LC50) values were calculated using GraphPad Prism 9 software [23]. The assay was conducted in triplicates and mean values were obtained [24].

## Results

### Antibacterial activity

#### Well diffusion assay

**Fig. 2:**
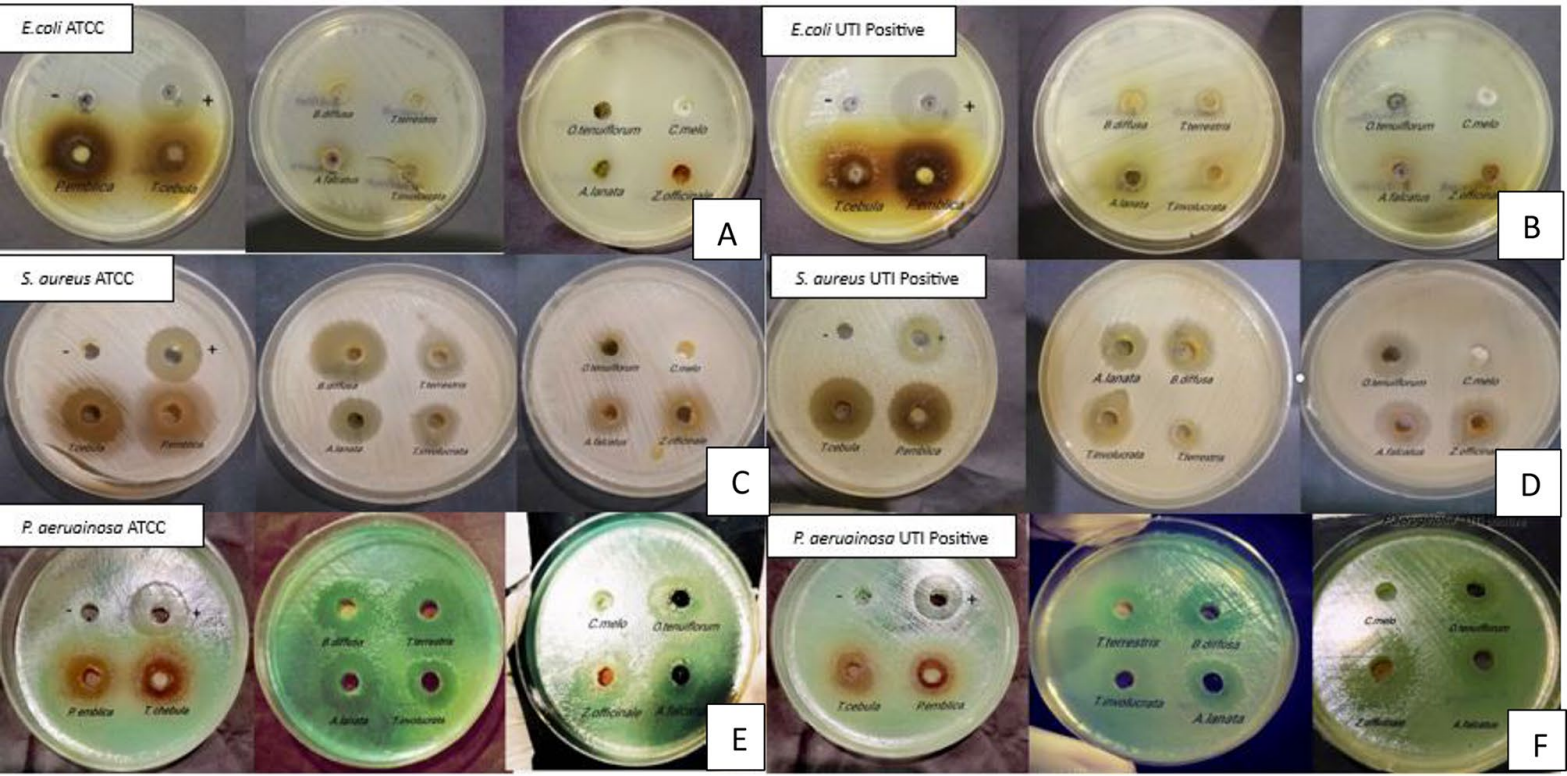
Well diffusion assay of selected medicinal plants agents (**A**) *E. coli ATCC25922*, (**B**) *E. coli* UTI positive strain, (**C**) *S. aureus* ATCC25923, (**D**) *S. aureus UTI* positive strain, (**E**) *P. aeruginosa* ATCC27853 and (**F**) *P. aeruginosa* UTI positive strain. The zone of inhibition was measured and each test was triplicated

Out of all the tested plant extracts, nine extracts including *P. emblica*,* T. chebula*,* B. diffusa*,* A. lanata*,* O. tenuiflorum Z. officinale*, *A. falcatus*,* T. terrestris*,* T. involucrata* showed antimicrobial activity against *E. coli* ATCC25922, *S. aureus* ATCC25923, *S. aureus* UTI Positive strain, *P. aeruginosa* ATCC27853, *P. aeruginosa* UTI Positive strain after 24 h of incubation (Fig. 2). *O. tenuiflorum and Z. officinale* did not show antimicrobial activity against the *Escherichia coli* UTI-positive strain and *C. melo* did not show any antimicrobial activity against all selected organisms. *P. emblica* (19 ± 1.2 mm) showed the highest activity against the *E. coli* ATCC25922 strain. Moreover, *P. emblica* (18 ± 0.6 mm) showed the highest activity against the *E. coli* UTI-positive strain. The highest inhibition was observed of *B. diffusa* (28 ± 0.6 mm) against *S. aureus* ATCC strain and *T. chebula* (25 ± 0.6 mm) showed the highest ZOI against *S. aureus* UTI Positive strain. *T. involucrata* (29 ± 1.0 mm) was observed to have the highest ZOI against *P. aeruginosa* ATCC27853 and *Z. officinale* (25 ± 0.6 mm) was observed to have the highest ZOI against *P. aeruginosa* UTI positive strain (Table 2).

**Table 2:** Mean ZOI and RPI of the selected plants against selected bacterial strains

Plant	*E.coli* ATCC25922		*E.coli* UTI Positive Strain		*S. aureus* ATCC25923		*S. aureus* UTI Positive Strain		*P*. *aeruginosa* ATCC27853		*P*. *aeruginosa* UTI Positive Strain	
	Mean ZOI (mm)	RPI (%)	Mean ZOI (mm)	RPI (%)	Mean ZOI (mm)	RPI (%)	Mean ZOI (mm)	RPI (%)	Mean ZOI (mm)	RPI (%)	Mean ZOI (mm)	RPI (%)
*P. emblica*	19±1.2	70%	18±0.6	82%	18±1.0	90%	24±1.5	150%	15±2.5	79%	16±0.6	89%
*T. chebula*	15±0.6	56%	15±0.6	68%	25±0.6	125%	25±0.6	156%	16±2.6	84%	14±0.6	78%
*T. terrestris*	10±1.5	37%	9±0.6	41%	16±0.6	80%	11±0.6	69%	17±2.0	89%	22±0.6	122%
*B. diffusa*	12±0.0	44%	10±0.0	45%	28±0.6	140%	22±0.6	137.50%	21±1.0	110.53%	23±0.6	128%
*T.involucrata*	10±0.0	37%	15±0.6	68%	15±0.6	75%	17±1.2	106.25%	29±1.0	153%	23±1.5	128%
*A.lanata*	12±0.6	44%	15±0.6	68%	17±0.6	85%	16±1.0	100%	21±0.6	110.53%	20±0.6	111%
*C. melo*	0±0.0	0%	0±0.0	0%	0±0.0	0%	0±0.0	0%	0±0.0	0%	0±0.0	0%
*O. tenuiflorum*	12±0.0	44%	0±0.0	0%	12±0.6	60%	12±1.5	75%	21±0.6	110.53%	19±1.5	105.56%
*Z. officinale*	13±1.2	48%	0±0.0	0%	16±2.0	80%	14±1.5	87.50%	19±1.0	100%	25±0.6	138.89%
*A. falcatus*	13±1.5	48%	15±0.0	68%	14±1.0	70%	14±0.0	87.50%	21±1.2	110.53%	21±0.0	116.67%
Negative Control (50% DMSO)	0.0±0.0		0.0±0.0		0.0±0.0		0.0±0.0		0.0±0.0		0.0±0.0	
Positive Control (Gentamycin)	27±1.5		22±2.5		20±0.6		16±0.6		19±1.5		18±0.6	

Values were triplicate and represented as mean ± SD. Zone of Inhibition concentration

#### Broth microdilution assay/ minimum inhibitory concentration (MIC) and minimum bacterial concentration (MBC)

The lowest MIC values were recorded for *P. emblica* against the *E. coli* ATCC strain and *T. involucrata* showed the lowest MIC against the *E. coli* UTI-positive strain, *B. diffusa* recorded the lowest MIC against *S. aureus* ATCC and *P. aeruginosa* ATCC strains. *T. chebula* showed the lowest MIC against *S. aureus* UTI positive strain and *A. lanata* showed the lowest MIC against *P. aeruginosa* UTI Positive strain (Table 3). Table 4 includes the MBC results for the selected plants.

**Table 3:** Mean MIC results of the medicinal plants against the bacterial strains

Plant	*E.coli* ATCC25922 (mg/ml)	*E.coli* UTI Positive Strain (mg/ml)	*S. aureus* ATCC25923 (mg/ml)	*S. aureus* UTI Positive Strain (mg/ml)	*P*. *aeruginosa* ATCC27853 (mg/ml)	*P*. *aeruginosa* UTI Positive Strain (mg/ml)
Positive control	0.781	6.25	0.39	6.25	3.125	3.125
*P. emblica*	0.024	25	0.39	0.39	1.563	3.13
*T. chebula*	12.5	25	0.39	0.098	12.5	12.5
*T. terrestris*	25	12.5	1.563	12.5	3.125	0.39
*B. diffusa*	12.5	6.25	0.195	0.781	0.098	6.25
*T.involucrata*	50	3.125	0.781	0.781	3.125	0.195
*A.lanata*	50	50	3.125	12.5	12.5	0.048
*O.tenuiflorum*	12.5	-	12.5	12.5	3.125	6.25
*Z. officinale*	12.5	-	0.39	0.781	6.25	25
*A.falcatus*	50	12.5	0.781	0.781	6.25	6.25

Values were triplicated

**Table 4:** Mean MBC results of the selected medicinal plants against selected bacterial strains

Plant	*E.coli* ATCC25922 (mg/ml)	*E.coli* UTI Positive Strain (mg/ml)	*S. aureus* ATCC25923 (mg/ml)	*S. aureus* UTI Positive Strain (mg/ml)	*P*. *aeruginosa* ATCC27853 (mg/ml)	*P*. *aeruginosa* UTI Positive Strain (mg/ml)
Positive control	12.5	25	12.5	25	12.5	6.25
*P. emblica*	12.5	25	12.5	12.5	25	50
*T. chebula*	50	50	12.5	12.5	50	50
*T. terrestris*	>50	>50	50	50	50	50
*B. diffusa*	50	25	6.25	25	6.25	50
*T. involucrata*	>50	50	25	25	25	12.5
*A.lanata*	>50	>50	50	25	50	50
*O. tenuiflorum*	50	-	50	25	25	25
*Z. officinale*	12.5	-	12.5	50	25	50
* A. falcatus*	>50	50	25	25	50	25

Values were triplicated

### Brine shrimp lethality assay (BSLA)

**Fig. 3:**
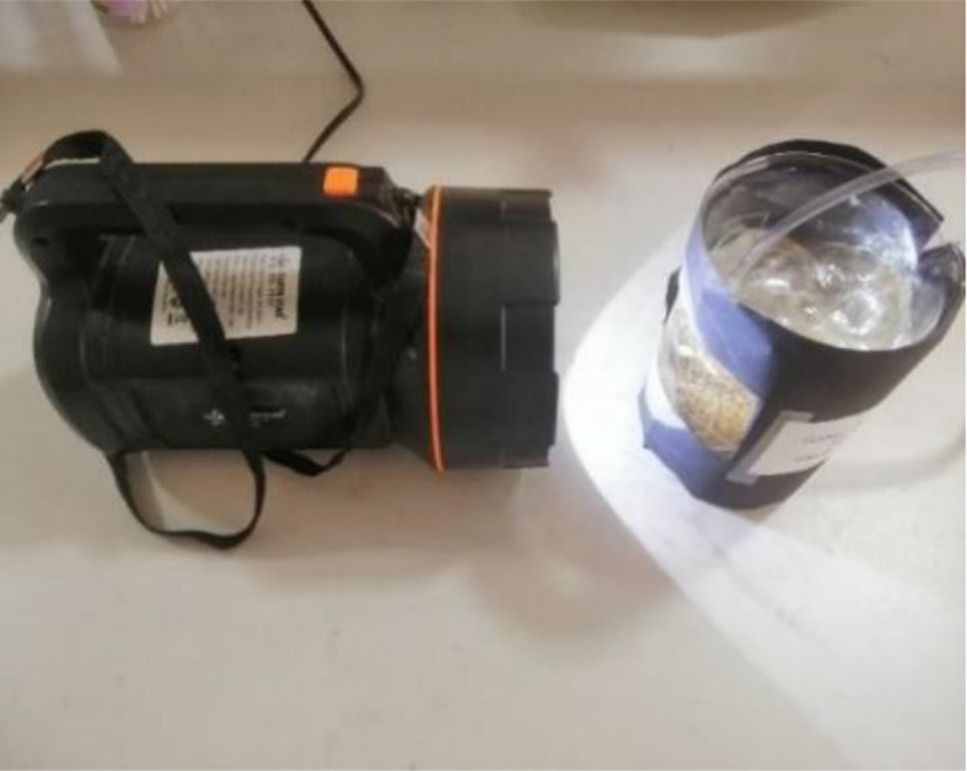
Setup used for BSLA. After turn on the light source and, oxygen supply *A. salina* organisms were hatched after 24–48 h

**Fig. 4:**
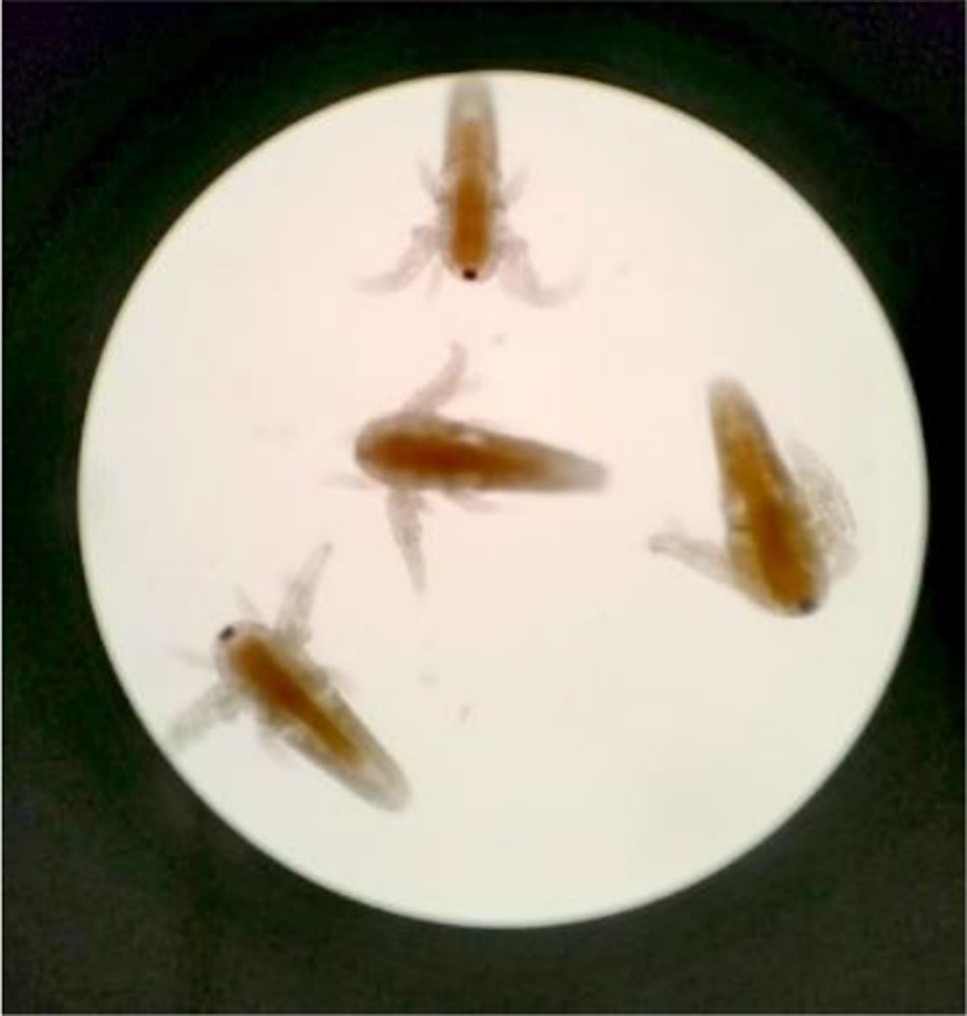
*A. salina* microscopic view under x40 light microscope

**Fig. 5:**
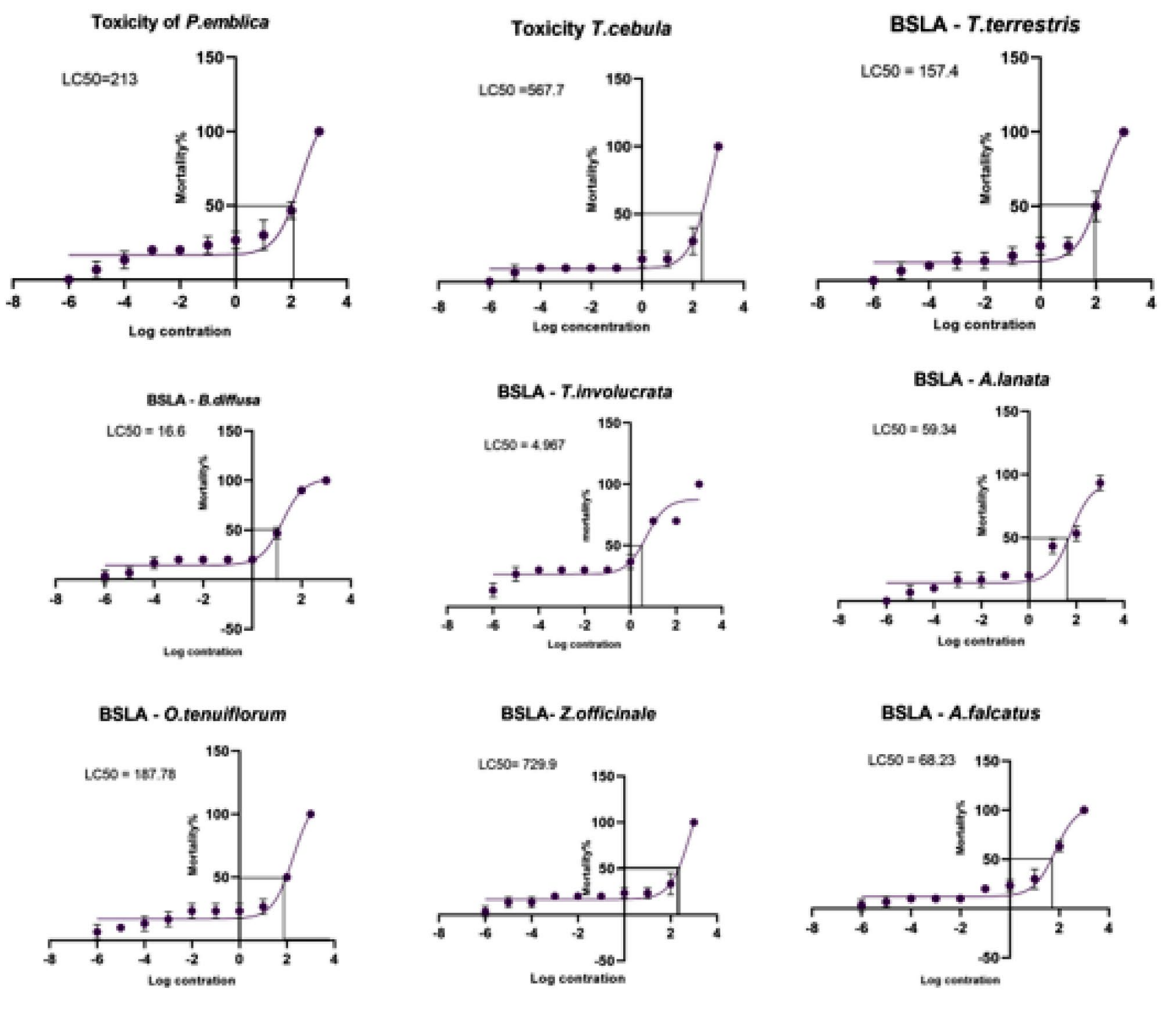
LC50 graphs of the selected medicinal plants in BSLA. All the graphs were created using GraphPad Prism 9 software

Once the BSLA was setup with a light source and an oxygen supply, we observed hatching of *A. salina* organisms within 24–48 h (Fig. 3). The number of live larvae was observed using the naked eye or using a light microscope (Fig. 4), LC50 (median lethal concentration) was calculated using GraphPad Prism 9 software according to a log concentration and mortality percentage. Table 5 included the BSLA LC50 values. The LC50 values compared with the Meyers’ toxicity scale (if LC50 < 1,000 µg/ml is considered toxic) [22, 25]. Figure 5 included the LC 50 graphs of the medicinal plants.

**Table 5:** BSLA results of the selected medicinal plants

Plant name	Log LC 50%(µg/ml)	LC 50% (µg/ml)
*P. emblica*	2.328	213
*T. chebula*	2.754	567.7
*T*,* terrestris*	2.197	157.4
*B. diffusa*	1.22	16.6
*T. involucrata*	0.6961	4.967
*A.lanata*	1.773	59.34
*C. melo*	1.468	29.41
*O.tenuiflorum*	2.273	187.7
*Z. officinale*	2.863	729.9
*A. falcatus*	1.834	68.23
Plant mixture	0.9390	8.69

LC50 values were calculated using GraphPad Prism 9 software. Values were triplicated

.

### Plant mixture preparation

The plant mixture was prepared based on the antimicrobial activity of each organism. Specifically, the plants were selected according to their antimicrobial efficacy against the chosen organisms. *P. emblica*,* T. chebula*,* and B. diffusa* exhibited higher mean zones of inhibition (ZOI) compared to the other plants. Consequently, plant material with higher and lower antimicrobial effect against all selected organisms were identified. Accordingly, *C. melo* did not show any antimicrobial activity against any of the selected organisms and therefore it was excluded from the final plant mixture. The final plant mixture was prepared using nine medicinal plants and dried plant powders of *P. emblica*,* T. chebula*,* T. terrestris*,* B. diffusa*,* T. involucrata*,* A. lanata*,* O. tenuiflorum*,* Z. officinale*,* A. falcatus* mixed according to the following ratios 1:1:4:1:4:4:2:2:3 based on their antimicrobial activities .

#### Plant mixture antimicrobial assays

**Fig. 6:**
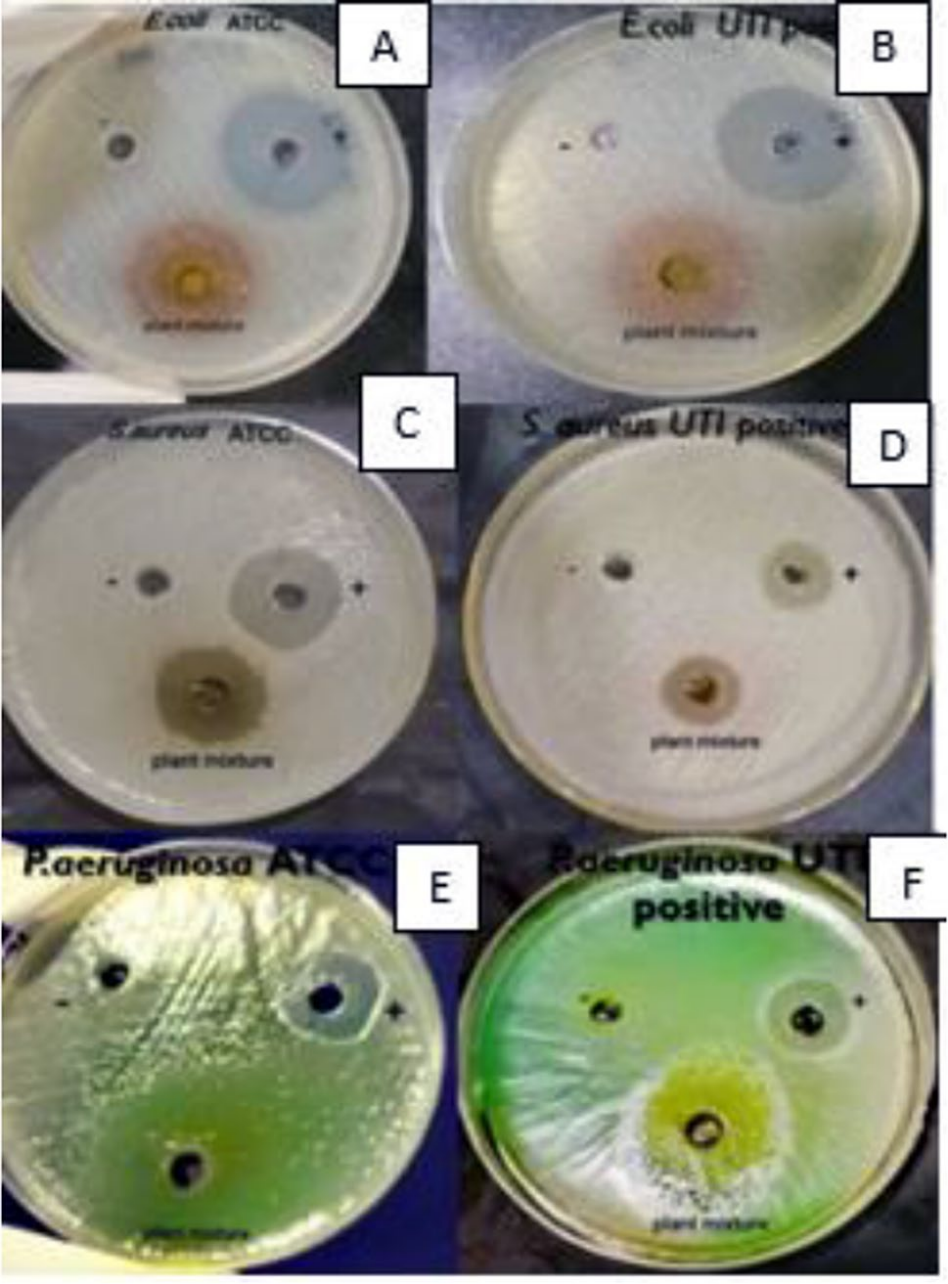
Well diffusion assay of plant mixture against (**A**) *E. coli ATCC25922*, (**B**) *E. coli* UTI positive strain, (**C**) *S. aureus* ATCC25923, (**D**). *S. aureus UTI* positive strain, (**E**) *P. aeruginosa* ATCC27853 and (**F**) *P. aeruginosa* UTI positive strain. The zone of inhibitions were measured

The plant mixture of 50 mg/ml showed antimicrobial activity against all six strains after 24 h of incubation (Fig. 6).

**Fig. 7:**
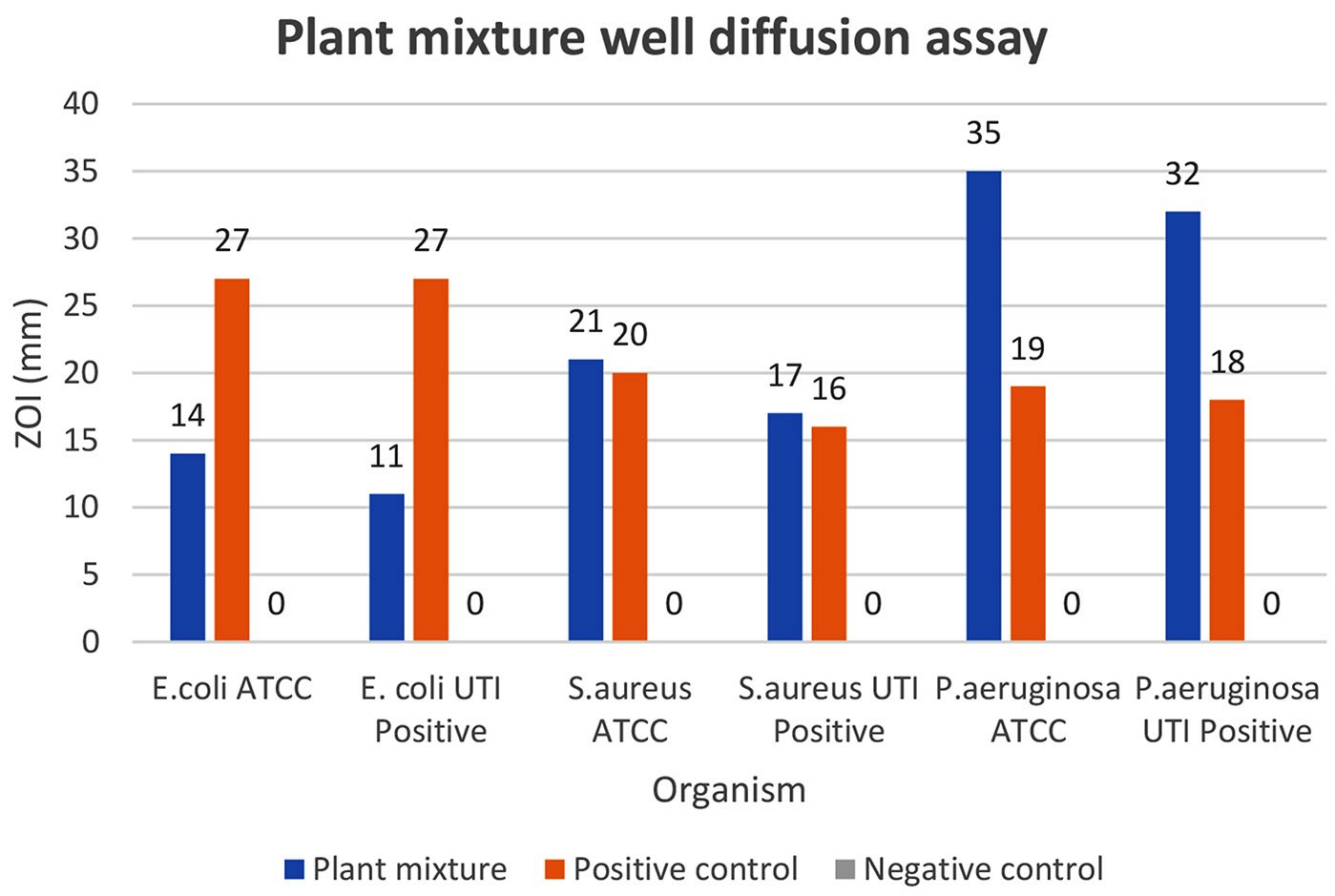
Well diffusion assay column chart of the plant mixture strains against selected bacterial strains

**Fig. 8:**
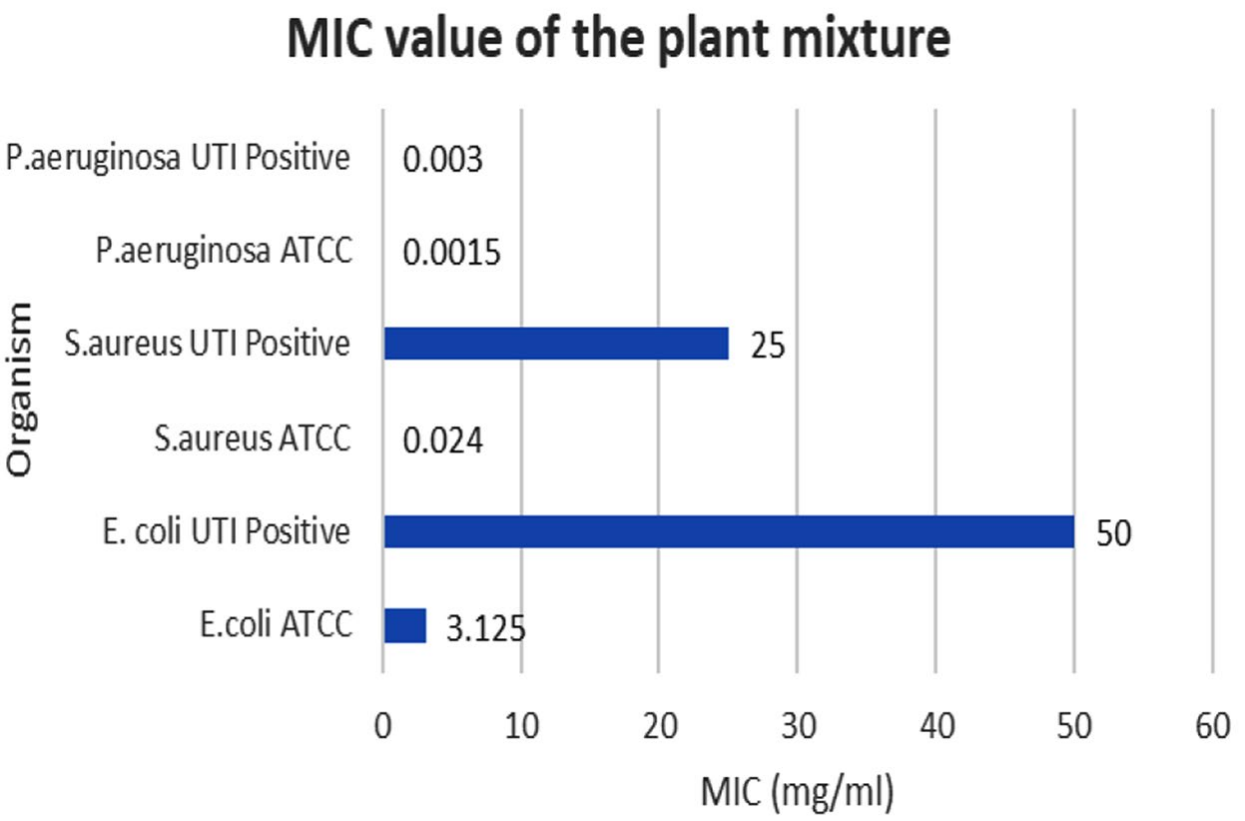
MIC bar chart of the plant mixture against selected bacterial strains

**Fig. 9:**
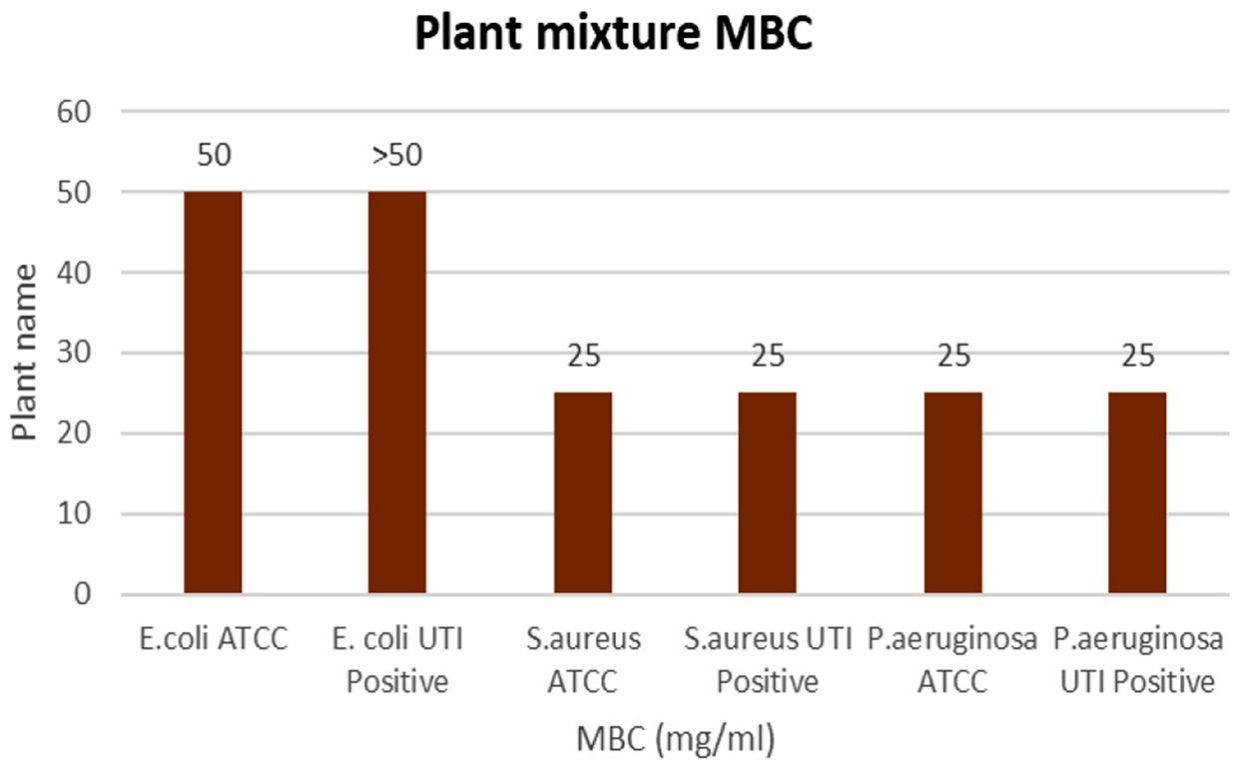
MBC column chart of the plant mixture strains against selected bacterial strains

The highest inhibition at 24 h was observed against *P. aeruginosa* ATCC27853 strain with a 35 ± 0.6 mm inhibitory zone. The lowest inhibition was shown against the *E. coli* UTI-positive strain with an 11 ± 1.2 mm inhibitory zone (Table 6) (Fig. 7). All relative percentage inhibitions observed with 50 mg/mL concentration were found to be higher than 40%. Both MIC and MBC values are depicted in Figs. 8 and 9.

**Table 6:** Mean ZOI and RPT of the plant mixture against selected bacterial strains

Plant	*E. coli* ATCC25922		*E. coli* UTI Positive Strain		*S. aureus* ATCC25923		*S. aureus* UTI Positive Strain		*P*. *aeruginosa* ATCC27853		*P*. *aeruginosa* UTI Positive Strain	
	Mean ZOI (mm)	RPI (%)	Mean ZOI (mm)	RPI (%)	Mean ZOI (mm)	RPI (%)	Mean ZOI (mm)	RPI (%)	Mean ZOI (mm)	RPI (%)	Mean ZOI (mm)	RPI (%)
Plant mixture	14±0.6	51.85%	11±1.2	40.74%	21± 0.6	95%	17±0.5	94.1	35±0.6	184.21%	32±0.6	177.78%
Positive Control (Gentamycin)	27±0.6		27±0.6		20± 0.6		16±0.6		19±0.6		18±0.6	
Negative Control (50% DMSO)	0±0		0±0.0		0± 0.0		0±0.0		0±0.6		0±0.0	

Zone of Inhibition - the mean values of triplicates

#### BSLA of the plant mixture

**Fig. 10:**
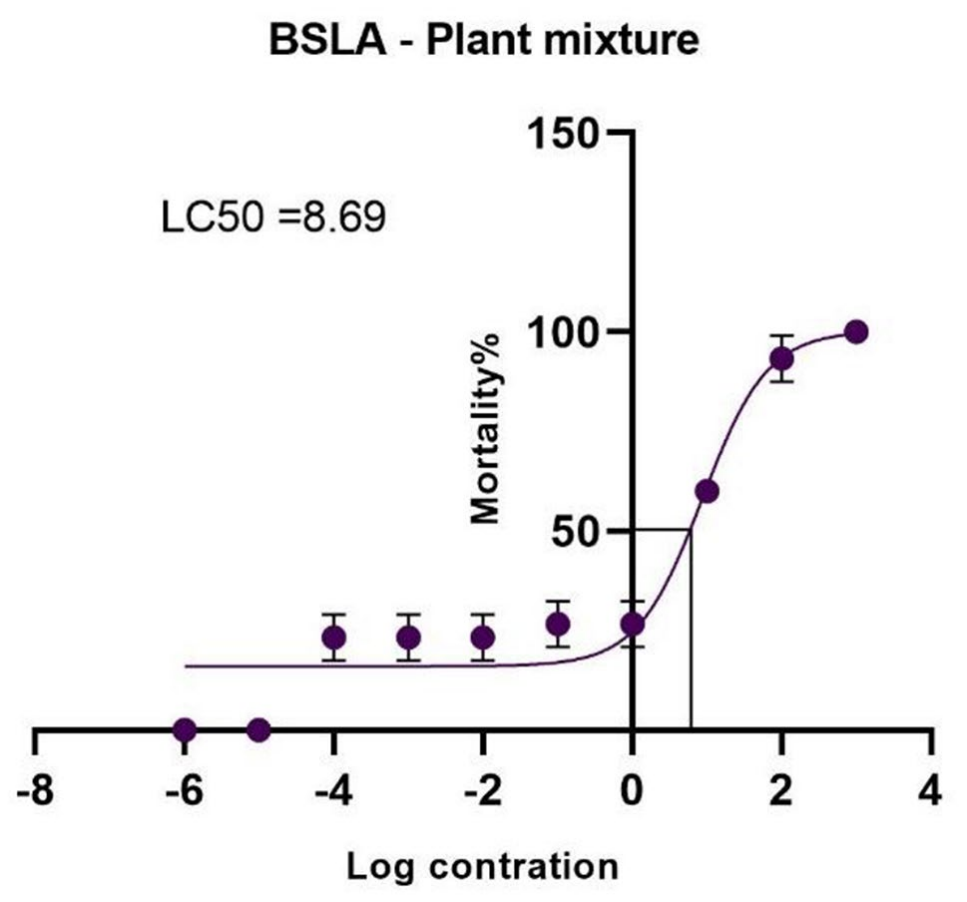
BSLA graph and LC50 value of the plant mixture

Plant mixture LC 50 value is 8.65 µg/ml (Fig. 10).

## Discussion

Medicinal plants have been used in traditional medicine to treat various infectious diseases since ancient times. In recent years, Sri Lankan traditional and Ayurvedic medicine has successfully excelled in treating patients with UTIs. The present study, investigated the antimicrobial properties of ten medicinal plants used in traditional and Ayurvedic medical practices for urinary tract infections (UTIs), focusing on their effectiveness against *E. coli*,* S. aureus*,* and P. aeruginosa* ATCC and pathogenic strains. Nine out of the ten selected plants exhibited antimicrobial activity against six organism strains, except *C. melo*. This highlights the potential of these medicinal plants in treating UTIs and demonstrates the importance of further exploring their therapeutic potential.

The present study used *E. coli*, *S. aureus* and *P. aeruginosa* strains that cause mainly complicated UTIs. The plant mixture was tested against these organisms’ ATCC and UTI-positive pathological strains. Antimicrobial effects of plants can be vary from country to country due to soil composition. The following plants *Phyllanthus emblica*,* Ocimum tenuiflorum*,* Terminalia chebula*,* Zingiber officinale*,* Tribulus terrestris*,* Tragia involucrate*,* Aerva lanata. Boerhavia diffusa* and *Asparagus falcatus* had an active profile [26].

According to the present study, *B. diffusa* methanolic roots extract is the best active extract identified for *S. aureus* ATCC25923 strain and *P. aeruginosa* ATCC27853 strains. *T. chebula* methanolic fruit extract is the best active extract identified for the *S. aureus* UTI-positive strain and *P. aeruginosa* UTI-positive strain. Additionally, *P. emblica* methanolic fruit extract is the best active extract identified for both *E. coli* ATCC25922 and *E. coli* UTI-positive strains. All plants except *C. melo* methanolic seed extract showed antimicrobial properties against six strains. They showed evident zones of inhibition in the agar well diffusion assay. The MIC values obtained from the present study indicated that the *P. emblica* methanolic fruits extract was more potent against *E. coli* ATCC25922; *B. diffusa* roots extract was more potent against *S. aureus* ATCC25923 and *P. aeruginosa* ATCC27853 strains. These results have some discrepancies in initial antimicrobial screening test results (agar well diffusion test). The differences in bacterial susceptibility between ATCC strains and pathogenic strains could be due to variations in the intrinsic tolerance of microorganisms. Bacteria can become resistant via genetic mutations or horizontal gene transfer. Mobile genetic elements such as plasmids and transposons are instrumental in transferring resistance genes horizontally [27]. Globally UTIs are treated with a variety of antibiotics, in this study Gentamycin was used as a reference antibiotic.

According to the previous literature, Silva et al. [28] showed the antimicrobial effects of the *C. melo* aqueous seed against these pathogens but in the present study, there were no antimicrobial properties observed. The present study’s results aligned with Narayanan et al. [6] findings for the methanolic extract of *A. lanata* against *S. aureus*, and *P. aeruginosa.* However, we observed a reduced antibacterial effect against *E.coli*. Narayanan et al. [6], reported the ethanolic extract showed poor or no antimicrobial effect against all organisms, while the ethyl acetate extract showed moderate activity than the methanolic extract against all the organisms. However bacterial strains were not specified [6]. We observed a higher antimicrobial effect for *B. diffusa* compared to the methanolic extract of Malhothra et al. [29]. The aqueous extract showed a lesser antimicrobial effect against the selected strains and no antimicrobial effect against *P. aeruginosa* [29]. *P. emblica* antimicrobial activity is similar to previous studies and not only methanolic extracts but also ethanolic extracts and essential oils showed good antimicrobial effects against *P. emblica* [30, 31]. Naik et al. [32] showed a significant effect against five organisms including *E. coli* and *P. aeruginosa* but their antibacterial effect was low compared to the present study. Rehman et al. [33], *T. chebula* did not show any antimicrobial effect against *E.coli.* However, the present study, *T. chebula*, showed antimicrobial effect for three selected organisms and showed the highest effect against *S. aureus* [33]. Sharma et al. 34, also supports the present study findings. The best antimicrobial effect was found in ethanol extract of *Z. officinale* and *T. chebula* against multi-drug resistance species, thus validating our finding [34]. Similar results were seen in Batoei et al. [35], Khalid et al. [36], and Ahamed et al. [37] where they have proven the antimicrobial effects against the *T. terrestris* plant. In this aspect our results were slightly different; it can be due to differences in selected parts and solvents. *T. involucrata* roots were used in the present study and it showed a significant effect against all selected organisms. Rajkumar et al. [38] used *T. involucrata* methanol, ethanol, aqueous, and chloroform extracts. All the extracts except aqueous showed a significant effect against *P. aeruginosa*. However, they used leaves and stems as the selected parts. Petroleum ether, acetone, chloroform, and aqueous extracts of *T. involucrata* roots did not show any antimicrobial effects against *E. coli* NCIM 2065 but leaves, stems and flowers showed the antimicrobial effects against *T. involucrata* [39]. Evidence was limited on the antimicrobial activity of *A. falcatus* roots, De Zoysa et al. [26] did not find any antimicrobial activity of *A. falcatus*, which we dispute as there was an antibacterial effect against three selected organisms and a higher antibacterial effect against both *P. aeruginosa* ATCC and pathogenic strains. Minor discrepancies could be attributed to different concentrations of solvents, and parts of the plants used.

The manual MIC method may produce false positive results due to difficulty in determining the MIC value using the naked eye. Therefore, incorporating quality control measures and adopting more reliable methods for MIC assay such as ELISA can minimize errors and improve the accuracy of the results. MBC results of the present study have discrepancies with MIC values. MIC values are in lower concentrations but most of the MBC values are closer to the stock solution concentration. Proper sterilization methods such as microfilters, freeze-drying can minimize variability in results. Following a systematic approach can ensure consistency, reproducibility, and desired outcomes.

When selecting plant material, it should be appropriate for the intended purpose and have desired properties. Considering factors such as the plant’s therapeutic potential, safety, availability, and compatibility with other ingredients, all the plants must be authenticated. Correct identification ensures the use of the intended plant and prevents potential misinterpretations or adverse effects. The plant preparation should be devoid of dirt, debris, or impurities.

The plant mixture was prepared according to the antimicrobial properties of each plant material. In this process, quality control measures were used to ensure the consistency and quality of the plant mixture. Quality control tests such as chromatography or fingerprinting techniques can be employed to assess the presence of specific compounds or marker substances. Storage and preservative methods should also be considered in further studies. In the present mixture, *P*. *emblica*,* T. chebula*,* T. terrestris*,* B. diffusa*,* T. involucrata*,* A. lanata*,* O. tenuiflorum*,* Z. officinale*,* A. falcatus* were added in the weight ratio of 1:1:4:1:4:4:2:2:3. Constituents of the combined extract were also tested individually for above six bacterial strains. *P. aeruginosa* ATCC and *P. aeruginosa* UTI-positive strains showed the highest antimicrobial effect with the plant mixture. However, the herbal formula showed satisfactory inhibition zones in agar well diffusion assay with 50 mg/mL stock solution of nine herbal plants except *C. melo* for all strains mentioned above.

Plant toxicity studies play a crucial role in ensuring the safety of herbal medicines, evaluating potential risks associated with plant consumption, and identifying plant toxins. In toxicity assessments, traditional methods such as animal studies are commonly used. The brine shrimp lethality assay (BSLA) is a widely used and cost-effective screening tool for assessing the toxicity of various substances, including plant extracts and compounds. It is a comparatively easy and cheap method, which provides the basic information useful to extend further cytotoxicity tests. This assay utilizes the sensitivity of brine shrimp (*Artemia salina*) nauplii to evaluate the lethality and potential cytotoxic effects of test samples [22]. Toxicity index values (LC50) of extracts were used to find out whether the extracts were toxic or non-toxic. If the LC50 value of an extract is greater than 1000 µg/ml, that extract is considered as a non-toxic extract [21, 24]. LC50 values of all samples were lower than 1000 µg/ml and are considered toxic according to the BSLA. If LC50 of a plant is between 0 and 100 µg/ml, it is considered as a highly toxic species. *T. involucrate* showed the lowest LD50 value (4.967 µg/ml) and plant mixture was the second most toxic substance (8.69 µg/ml). While the BSLA offers a rapid and cost-effective screening tool, it is important to consider certain limitations. The assay’s reliance on a single species (brine shrimp) as a toxicity indicator may not fully represent the complexity of mammalian systems. Additionally, factors such as variations in the nauplii quality, temperature, and salinity can influence the assay results. Hence, the BSLA should be complemented with additional toxicity studies and mechanistic investigations to obtain a comprehensive understanding of plant toxicity.

## Conclusion

Based on the present study methanolic extracts of the tested medicinal plants except *C. melo* seeds extract showed the antibacterial activity against *E. coli*,* S. aureus* and *P. aeruginosa*. Most exhibited higher antibacterial activity against *P. aeruginosa* compared to the other organisms. The results of the present study evidently support the traditional usage of these plants for the treatment of UTIs. It pave the way to the novel medicine and treatment development. Furthermore, the results obtained in this study may justify the all the species including plant mixture had antimicrobial activity and showed significant toxic effects on brine shrimps. Hence, need future toxicity assays to confirm their actual toxicity and dosage. Therefore, further investigations need to be carried out before recommending these medicinal plants as a treatment for UTI.

## Data Availability

The datasets during and/or analyzed during the current study are available from the corresponding author on reasonable request.

## Abbreviations

ABST: Antibiotic Susceptibility Testing
*A. falcatus*: *Asparagus falcatus*
*A. lanata*: *Aerva lanata*
*A. salina*: *Artemia salina*
*B. diffusa*: *Boerhavia diffusa*
BMD: Broth Micro dilution
*BSLA*: *Brine Shrimp Lethality Assay*
*C. melo*: *Cucumis melo*
CLSI: Clinical and Laboratory Standards Institute
DMSO: Dimethyl sulfoxide
*E. coli*: *Escherichia coli*
ELISA: Enzyme Linked Immunosorbent Assay
MBC: Minimum Bactericidal Concentration
MHA: Muller Hinton Agar
MHB: Muller Hinton Broth
MIC: Minimum Inhibitory Concentration
MLT: Medical Laboratory Technician
NA: Nutrient Agar
*O. tenuiflorum*: *Ocimum tenuiflorum*
*P. aeruginosa*: *Pseudomonas aeruginosa*
*P. emblica*: *Phyllanthus emblica*
RPI: Relative Percentage of Inhibition
*S. aureus*: *Staphylococcus aureus*
SD: Standard Deviation
*T. chebula*: *Terminalia chebula*
TI: Therapeutic Index
*T. involucrate*: *Tragia involucrata*
*T. terrestris*: *Tribulus terrestris*
UPEC: Uropathogenic *Escherichia coli*
UTI: Urinary Tract Infection
WHO: World Health Organization
*Z. officinale*: *Zingiber officinale*
ZOI: Zone of Inhibition

## Declarations

### Plant authentication

All selected ten plants from the Botany Division, Bandaranayake Memorial Ayurvedic Research Institute, Nawinna, Maharagama, Sri Lanka.
